# Decreased expression of GST pi is correlated with a poor prognosis in human esophageal squamous carcinoma

**DOI:** 10.1186/1471-2407-10-352

**Published:** 2010-07-05

**Authors:** Zhihui Wang, Wei He, Guanrui Yang, Junsheng Wang, Zhong Wang, Jahn M Nesland, Ruth Holm, Zhenhe Suo

**Affiliations:** 1 Department of Oncology, The First Affiliated Hospital of Zhengzhou University, Medical College of Zhengzhou University, Zhengzhou, China; 2 Department of Pathology, Oslo University Hospital, Ullernchausseen 70, Oslo, Norway; 3 Henan Institute of Medical Science, Medical College of Zhengzhou University, Zhengzhou, China; 4 Department of Oncology, Anyang Tumor Hospital, Henan, China

## Abstract

**Background:**

Glutathione S-transferase pi (GST pi) is a subgroup of GST family, which provides cellular protection against free radical and carcinogenic compounds due to its detoxifying function. Expression patterns of GST pi have been studied in several carcinomas and its down-regulation was implicated to be involved in malignant transformation in patients with Barrett's esophagus. However, neither the exact role of GST pi in the pathogenesis nor its prognostic impact in squamous esophageal carcinoma is fully characterized.

**Methods:**

Immunohistochemistry was used to investigate GST pi expression on 153 archival squamous esophageal carcinoma specimens with a GST pi monoclonal antibody. Statistic analyses were performed to explore its association with clinicopathological factors and clinical outcome.

**Results:**

The GST pi expression was greatly reduced in tissues of esophageal carcinomas compared to adjacent normal tissues and residual benign tissues. Absent of GST pi protein expression in cytoplasm, nuclear and cytoplasm/nucleus was found in 51%, 64.7% and 48% of all the carcinoma cases, respectively. GST pi deficiency in cytoplasm, nucleus and cytoplasm/nucleus was significantly correlated to poor differentiation (*p *< 0.001, *p *< 0.001 and *p *< 0.001, respectively). UICC stage and T stage were found significantly correlated to negative expression of GST pi in cytoplasm (*p *< 0.001 and *p *= 0.004, respectively) and cytoplasm/nucleus (*p *= 0.017 and *p *= 0.031, respectively). In univariate analysis, absent of GST pi protein expression in cytoplasm, nucleus and cytoplasm/nucleus was significantly associated with a shorter overall survival (*p *< 0.001, *p *< 0.001 and *p *< 0.001, respectively), whereas only GST pi cytoplasmic staining retained an independent prognostic significance (*p *< 0.001) in multivariate analysis.

**Conclusions:**

Our results show that GST pi expression is down regulated in the squamous esophageal carcinoma, and that the lack of GST pi expression is associated with poor prognosis. Therefore, deficiency of GST pi protein expression may be an important mechanism involved in the carcinogenesis and progression of the squamous esophageal carcinoma, and the underlying mechanisms leading to decreased GST pi expression deserve further investigation.

## Background

Esophageal cancer (EC) ranks the third among most common cancer of the digestive tract and the seventh leading cause of cancer-related deaths worldwide [1,2]. With new cases accounting for nearly half new cases of the world per year, China is among the highest incidence areas [3]. EC is usually diagnosed at late stages with a five-year survival rate of only 5-10 percent [3,4]. Surgical resection is believed to offer the best chance for long-term survival compared to other therapies such as radio- and chemotherapy, used alone or in combination as adjuvant treatments [3,5-8]. However, surgical resection is often unavoidably followed by considerable compromised life-quality. Therefore, individualized therapy which benefits patients with the highest treatment efficiency yet the least morbidity is increasing stringent for treatment. To pave the way for it, it is important to identify prognostic markers and predictors of significance value in this tumor [9].

Glutathione S-transferases (GSTs), a supergene family with at least four distinct isoforms (α, μ, π, θ) identified in human, are involved in the metabolism of xenobiotic compounds in the phase II detoxification [10-12]. They are capable of converting a variety of electrophilic and hydrophobic compounds into more soluble, more easily excretable compounds through catalyzing them in conjunction with glutathione [10]. As numerous potentially toxic carcinogenic compounds, being electrophilic and hydrophobic, are detoxified in this way, GSTs is believed to play an important role in cancer prevention [13,14]. Down-regulation of GSTs has been reported as an increased risk for developing gastric, colorectal, and lung cancer [15-17]. Decreased GST enzyme activity in the gastrointestinal track is implicated with a raised tumor incidence [2].

GST pi, the predominant isoform in the normal squamous esophagus epithelium [18], is present in a wide range of normal human tissues [18,19], as well as in various malignant tumors of urinary, digestive, and respiratory tracts [20-24]. No consensus has been achieved yet regarding to the association between GST pi expression and malignant transformation. Some studies suggest an increased expression of GST pi as an indicator for premalignant and malignant changes [25,26]; Whereas, in others, GST pi expression is indicated to be a marker of carcinogen exposure in the upper aerodigestive tract [27-30]. Meanwhile, in some studies, loss of GST pi expression is suggested as a phenotype associated with carcinogenesis [31,32].

As to the alternation of GST pi in development of esophageal carcinoma, several studies have been performed on Barrett's metaplasia and adenocarcinoma with results suggesting deficiency of GST pi may contribute to an increased cancer risk [2,33,34]. However, limited knowledge is available in terms of GST pi alternation in squamous esophageal carcinoma, as well as its connection with clinical parameters. Therefore, in the present study, we report results of an immunohistochemical survey of GST pi in 153 squamous esophageal carcinoma cases with a long term follow-up. Our study confirms a down-regulated GST pi expression in this type of tumor, and demonstrates the deficiency of GST pi protein expression is significantly associated with a shorter overall survival.

## Methods

### Patient materials

One hundred and fifty-three patients composed by 93 men and 60 women with esophageal squamous cell carcinoma, whom underwent potentially curative surgery during 1989-1994 at Anyang Tumor Hospital, Henan, China, were enrolled in this retrospective study. The median age at diagnosis was 56.4 years (range 33-73 years). No preoperative chemotherapy and radiotherapy were given. All tumors were staged according to International Union against Cancer (UICC) 2003 Classification. One hundred (65.4%) cases were classified as stage II and 53 (34.6%) cases as III. All patients were followed up until death or 31. May, 2004. Ninety-seven (63.4%) patients died of esophageal cancer. The median follow-up time for all patients was 90 months (range 1-155 months). The follow-up and data analyses were performed by researchers from both sides of this international cooperation project, Anyang Tumor Hospital, China and The Norwegian Radium Hospital, Norway. Patients' information included tumor size, TNM staging, pathologic grade, demographic data and mortality. Study approval was given by the Regional Committee for Medical Research Ethics in Norway.

Specimens removed from surgery were fixed in formalin, processed and embedded in paraffin blocks for diagnosis and research use. Histological specimens were reviewed by two of the co-authors (Z.S and J.M.N) who had no access to clinical information. The detailed description as to the tumor characteristics was listed in Table 1.

**Table 1:** GST pi immunostaining in relation to clinicopathological variables

Variables	Total	Cytoplasm			Nucleus			Cytoplasm and Nucleus		
				
	N (%)	Negative (%)	Positive	*p*^1^	Negative (%)	Positive	*p*^1^	Negative (%)	Positive	*p*^1^
Age				0.019			0.405			0.060
<51	48 (31)	18 (38)	30		28 (58)	20		19 (40)	29	
51-60	52 (34)	34 (65)	18		37 (71)	15		32 (62)	20	
>60	53 (35)	26 (49)	27		34 (64)	19		23 (43)	30	
gender				0.185			0.302			0.136
Male	93 (61)	43 (46)	50		57 (61)	36		40 (43)	53	
Female	60 (39)	35 (58)	25		42 (70)	18		34 (57)	26	
Histological grade				<0.001			<0.001			<0.001
Well	53 (35)	13 (25)	40		22 (42)	31		12 (23)	41	
Moderate	60 (39)	38 (63)	22		45 (75)	15		36 (60)	24	
Poor	40 (26)	27 (68)	13		32 (80)	8		26 (65)	14	
Location				0.232			0.929			0.995
Upper	14 (9)	5 (36)	9		9 (64)	5		7 (50)	7	
Middle	101 (66)	47 (47)	54		67 (66)	34		49 (49)	52	
Lower	35 (23)	21 (60)	14		22 (63)	13		17 (49)	18	
Missing	3 (2)	2	1		2	1		2	1	
Size				0.238			0.973			0.843
≤30 mm	25 (16)	11 (44)	14		17 (68)	8		11 (44)	14	
31-60 mm	105 (69)	59 (56)	46		70 (67)	35		53 (51)	52	
>60 mm	14 (9)	5 (36)	9		9 (64)	5		7 (50)	7	
Missing	9 (6)	3	6		4	5		3	6	
Lymph node metastasis				0.090			0.727			0.398
-	99 (65)	45 (46)	54		63 (64)	36		45 (46)	54	
+	54 (35)	33 (61)	21		36 (67)	18		29 (54)	25	
UICC stage				<0.001			0.377			0.017
II	100 (65)	40 (40)	60		62 (62)	38		41 (41)	59	
III	53 (35)	38 (71)	15		37 (70)	16		33 (62)	20	
T stage				0.004			0.126			0.031
I+II	44 (29)	14 (32)	30		23 (52)	21		14 (32)	30	
III	100 (65)	57 (57)	43		70 (70)	30		54 (54)	46	
IV	9 (6)	7 (78)	2		6 (67)	3		3 (33)	6	

^1^Pearson chi-square

### Tissue array method

Multi-tissue array paraffin blocks were produced by a MTA-1 manual tissue arrayer (Beecher Instruments Inc., Sun Prairie, WI, U.S.A), before 5 μm paraffin sections were cut and underwent the Hematoxyline and Eosin (H&E) staining. After evaluation, two representative tumor areas and one stroma area on each sample were selected, respectively. The chosen regions were then removed from the original paraffin block to a recipient paraffin block by using the hollow-cored needles with the core-diameter of 0.6 mm. By this way, the pin-picked chosen tissues were arrayed on the recipient paraffin block. Finally, five μm sections made from those recipient paraffin blocks were cut and mounted on the charged Super-Frost Plus glass slides, and ready for immunohistochemistry analysis after pre-dried at 60ÂºC in an oven for 2-4 hours. For samples on which the representative areas were failed to choose for tissue array, five μm sections from the whole tissue paraffin blocks underwent immunohistochemical analysis as well.

### Immunohistochemical method

Dako EnVision™ + System, Peroxidase (DAB) (K4007, Dako Corporation, CA, and U.S.A) was employed for immunostaining. Sections were first deparaffinized in xylene and microwaved in 10 mM citrate buffer pH 6.0 to unmask the epitopes, and then treated with 0.3% hydrogen peroxide (H_2_O_2_) for 5 min to block the endogenous peroxidase. Afterwards, monoclonal glutathione-S-transferanse pi antibody (clone 353-10, 1:50, from Acris Antibodies GmbH, Germany) was applied on the sections for 30 min at room temperature, followed by an incubation with the horzeradish peroxidase (HRP) labeled polymer conjugated to goat anti-mouse IgG for 30 min at room temperature. Sections were then incubated with 3'3-diaminobenzidine tetrahydrochloride (DAB) for 10 min, and counterstained with hematoxylin, dehydrated and mounted in Diatex before evaluation.

Immunostaining of each section was semiquantitatively scored for both intensity (1, absent/weak; 2, moderate; 3, strong) and extent of staining (percentage of the positive tumor cells: 1, < 10%; 2, 10-50%; 3, > 50%). The scoring results of intensity and extent were multiplied to give a composite score ranging from 1 to 9 for each section. Examination of immunostaining was performed by two independent observers (Z.W and Z.S) with no knowledge of patients outcome. All discordant scores were reviewed until final agreements were obtained.

### Statistical analyses

The associations between GST pi protein expression and clinicopathologic variables were evaluated by the Person χ^2 ^test. The Kaplan - Meier method were employed to estimate the survival rate. A Cox proportional hazards regression model was formed to perform multivariate evaluation of survival rates, after the fulfillment of proportional hazard assumption of variables was evaluated by STATA statistical software package (Stata 10.0, collage station, TX). The calculation was performed by usage of the SPSS 16.0 statistical software package (SPSS, Chicago, IL), and *p *â‰¤ 0.05 was considered as statistical significance.

## Results

### Frequency of GST pi protein expression

**Figure 1:**
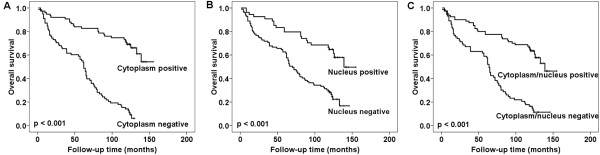
**Expression of GST pi protein in squmous esophagus epithelium**. Expression of GST pi protein in normal squamous esophagus epithelium (A) and (B). Expression of GST pi protein in esophageal squamous carcinoma with positive immunostaining in cytoplasm and negative in nucleus (C). Expression of GST pi protein in esophageal squamous carcinoma with negative immunostaining in cytoplasm and positive in nucleus (D). Expression of GST pi protein in esophageal squamous carcinoma with positive immunostaining in both cytoplasm and nucleus (E). Expression of GST pi protein in esophageal squamous carcinomas with negative immunostaining in both cytoplasm and nucleus (F). Original magnification: × 400 (A, B); × 600 (C-F); × 200 (pictures at lower right corner of C-F).

The majority of cases contained adjacent normal tissues and residual benign tissues, on which strong positive staining presented and served as the internal control for both cytoplasm and nucleus. Immunostaining in adjacent normal tissues and residual benign tissues was found in parabasal, middle and top layers of the esophageal epithelium (Figure 1a and 1b).

In esophageal carcinomas, positive immunostaining in either cytoplasm (Figure 1c), or nucleus (Figure 1d), or cytoplasm/nucleus (Figure 1e) was discoved in 75 (49%), 54 (35.3%), and 79 (51.6%) cases, respectively. Negative immunostaining for GST pi in either cytoplasam (Figure 1d), or nucleus (Figure 1c), or both cytoplasma and nucleus (Figure 1f) was found in 78 (51%), 99 (64.7%), and 74 (48.4%) cases, respectively. The summarized GST pi immunostaining score was listed in Table 2.

**Table 2:** Immunostaining results for GST pi

Score	Cytoplasm		Nucleus		Cytoplasm/nucleus	
			
	n	(%)	n	(%)	n	(%)
0	78	(51.0)	99	(64.7)	74	(48.4)
3	54	(35.3)	43	(28.1)	58	(37.9)
6	21	(13.7)	10	(6.5)	17	(11.1)
9	0	(0)	1	(0.7)	4	(2.6)
Total	153	(100.0)	153	(100.0)	153	(100.0)

### GST pi immunostaining in relation to clinicopathological parameters and patients survival

GST pi immunostaining status in relation to clinicopathological parameters was summarized in Table1. Negative expression of GST pi in cytoplasm, nucleus and cytoplasm/nucleus was significantly correlated to high histological grade (*p *< 0.001, *p *< 0.001 and *p *< 0.001, respectively). UICC stage and T stage were found significantly correlated to negative expression of GST pi in cytoplasm (*p *< 0.001 and *p *= 0.004, respectively) and cytoplasm/nucleus (*p *= 0.017 and *p *= 0.031, respectively). No significant associations were found between GST pi expression levels and age, gender, location, tumor size and lymph node metastasis.

**Figure 2:**
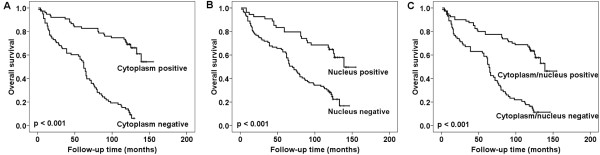
**Survival curves using the Kaplan-Meier method**. Kaplan-Meier curves drawn for the entire series (n = 153) based on GST pi protein expression levels in cytoplasm (*p *= 0.001) (A). Kaplan-Meier curves drawn for the entire series (n = 153) based on GST pi protein expression levels in nucleus (*p *< 0.001) (B). Kaplan-Meier curves drawn for the entire series (n = 153) based on GST pi protein expression levels in cytoplasm/nucleus (*p *= 0.001) (C).

In evalution of proportional hazard assumption of variables, it was found that variables including UICC stage, T stage and GST pi staining in cytoplasm, nucleus and cytoplasm/nucleus were qualified for such analysis, and all survival curves did not cross substantially. Histological grade did not fulfill proportional hazard assumption, but was used in multivariate analysis as a strata variate. In univariate analysis, high UICC stage (*p *< 0.001), high T stage (*p *< 0.001 and *p *= 0.001), and GST pi staining in cytoplasm, nucleus and cytoplasm/nucleus (*p *< 0.001, *p *< 0.001 and *p *< 0.001 respectively) were associated with poor overall survival (Figure 2). In multivariate analysis with histological grade as a strata variate, only T stage and GST pi cytoplasmic staining retained independent prognostic significance (*p *< 0.001, *p *< 0.001 and *p *< 0.001, respectively) (Table 3).

**Table 3:** Relative risk (RR) of dying from esophageal squamous carcinoma

Variables	Univariate analysis			Multivariate analysis		
		
	RR	95% CI^a^	*p*	RR	95% CI^a^	*p*
UICC stage	3.37	2.24-5.07	<0.001	-	-	-
T stage (III/I+ II)	3.46	1.86-6.44	<0.001	3.94	2.22-7.01	<0.001
T stage (IV/I + II)	4.85	1.98-11.91	0.001	8.83	3.73-20.90	<0.001
GSTpi cytoplasmic staining	5.63	3.50-9.06	<0.001	4.80	2.94-7.84	<0.001
GSTpi nucleus staining	2.92	1.80-4.73	<0.001	-	-	-
GSTpi cytoplasmic/nucleus staining	3.95	2.56-6.11	<0.001	-	-	-

^a^95% confidence interval

## Discussion

Previously, GST pi expression in tissue and serum was suggested as a cancer marker in several studies [10,26], with results showing inconsistent GST pi expression patterns in various carcinomas. In gastric cancer, an increased serum GST pi was hypothesized to correlate to the advanced stage, and its expression in tissue was found inversely correlated to survival [35]. In precancerous foci of colorectal polyps, an increased GST pi expression presented in the high grade adenoma [36]. However, in prostate adenocarcinoma, down-regulation of GST pi was observed and the loss of GST pi expression was regarded as a phenotype associated with malignant transformation [31,37].

In esophageal carcinomas, many studies showed that down-regulation of GST pi expression was involved in malignant progression from Barrett's esophagus to esophageal adenocarcinoma [2,34]. The decreased GST pi expression was observed in Barrett's epithelium [2,34,38], and lower GST pi mRNA levels were detected by Northern blot analysis and measurements of enzymatic activity [38-40]. These studies anticipated that the absence of this detoxifying enzyme may play an important role in development and progression of esophageal carcinoma [38,41]. However, a study on limited samples (53 samples from 19 patients) by Chandra et al. [2] argued that high GST pi expression connoted a poor prognosis. At the same time, the authors failed in finding statistical significant difference to the disease-free survival [2].

In our present study, down-regulated GST pi was observed in squamous esophageal carcinomas, a finding which is rather consistent with the esophageal carcinoma studies by Huang et al. [42] and Fu et al. [43]. Both Huang's and our study find that loss of GST pi expression is associated with clinicopathological factor of high histological grade. Furthermore, our results also demonstrate that the reduced GST pi expression is correlated to the overall survival, indicating that GST pi down-regulation is not only associated with esophageal tumorigenesis, a process with a variety of genetic and epigenetic alterations [34,38,44], but also with esophageal tumor progression.

GST pi polymorphism has been suggested to be involved in alternation of GST pi enzyme activity. Van et al. [45] showed that the reduced GST pi enzyme activity in Barrett's esophagus was attribute to the expression of GST pi b, a genotype encoding GST pi enzyme with a less activity, compared with GST pi a. Compton and coworkers [40] suggested that down-regulation of GST pi was second to the decreased GST pi mRNA level, which happened in transcription step due to gene interactions [38]. In addition, decreased level of GST pi was implied to connect with the epigenetic alternation, resulting in the transcriptional silence. Hypermethylation of CpG islands within promoter regions has been found in several genes, and has been implied to be responsible for down-regulation of their protein products [46-48]. Hypermethylation of GST pi gene, although happened at a low frequency, has been reported in esophageal adenocarcinomas [44]. Potential mechanisms accounting for down-regulation of GST pi in malignant transduction in Barrett's esophagus may take place in squamous esophageal carcinoma as well. We speculate that epigenetic alterations may happen in squamous esophageal carcinomas and results in a decreased GST pi expression in this type of tumor.

Limited knowledge is known whether an increased risk of cancer development is secondary to GST pi down-regulation or not, but some studies on esophagus have suggested that imbalance between redox and GST enzyme might get involved [2]. According to the study of Chandra et al. [2], redox molecular species may play double-edged roles. On one side, they could kill tumor cells by inducing cell apoptosis and/or through other mechanisms. On the other side, due to the redox-mediated damages on DNA molecules, they could initiate a cascade of mutational events which promoted the development or progression of malignancy.

Studies on NOâˆ™, a redox molecular, have found that upper aerodigestive track malignancies strongly expressed the enzymatic machinery necessary to generate NOâˆ™, in spite of its known physiologic roles on regulation of vascular blood flow and an assistance on killing infectious and malignant cells [49,50]. The prevalence expression of NOâˆ™ in tumor cells indicated a potential high concentration of NOâˆ™ in microenvironment of tumor [1,33,51] raising concerns of mutagenesis due to breaking up the double-strand structure of DNA molecules [52]. However, effects of high NOâˆ™ levels can be counteracted by glutathione [53]. GST enzymes, by catalyzing glutathione to nucleophilic compounds, provide a key biochemical sink for free radicals and highly reactive molecules [2]. Lack of GST expression may lead to accumulation of redox-mediated DNA damages in cells, contributing to genome instability as a result of an imbalance between GST enzymes and redox species.

As to development and progression of squamous esophageal carcinoma, we speculate that lack or loss of GST pi protein expression may predispose a normal cell to undergo further genetic alternations, raising risks of malignant changes ultimately and even tumor progression.

## Conclusions

The present study, by using immunohistochemistry on 153 cases, confirms that the expression of GST pi is down-regulated in squamous esophageal epithermal carcinomas and significantly associated with poor overall survival. Deficiency of GST pi protein expression may be an important mechanism involved in the carcinogenesis and progression of esophageal squamous carcinomas. Further studies are deserved to explore the underlying mechanisms leading to decreased GST pi expression in this type of tumor.

## Competing interests

The authors declare that they have no competing interests.

## Authors' contributions

ZW participated in the design of the study, carried out the immunohistochemical analysis, statistical and data analysis and draft the manuscript. WH participated in the design of the study, interpretation of data and manuscript revising. GY, JW and ZW were involved in the project design, collection of clinical data, interpretation of data and manuscript revising. JMN revised the manuscript critically. RH participated in statistical and data analysis and helped to draft the manuscript. ZS participated in the design of the study, statistical and data analysis and helped to draft the manuscript. All authors read and approved the final manuscript.

## Pre-publication history

The pre-publication history for this paper can be accessed here:

http://www.biomedcentral.com/1471-2407/10/352/prepub
